# Hybrid convergent ablation versus endocardial catheter ablation for atrial fibrillation: a systematic review and meta-analysis of randomised control trials and propensity matched studies

**DOI:** 10.1186/s13019-022-01930-7

**Published:** 2022-08-13

**Authors:** Aditya Eranki, Ashley R. Wilson-Smith, Michael L. Williams, Campbell D. Flynn, Con Manganas

**Affiliations:** 1 Department of Cardiothoracic Surgery, St George Hospital, Gray Street Kogarah, Sydney, Australia; 2 Department of Cardiothoracic Surgery, John Hunter Hospital, Lookout Road, Newcastle, Australia; 3 The Collaborative Research (CORE) Group, Macquarie University, Sydney, Australia; 4 Department of Cardiothoracic Surgery, Dunedin Hospital, Great King Street, Dunedin, Otago New Zealand

**Keywords:** Hybrid ablation, Convergent procedure, Atrial fibrillation, Systematic review

## Abstract

**Introduction:**

Atrial fibrillation (AF) is the most common arrhythmia. Hybrid convergent ablation (HCA) is an emerging procedure for treating longstanding AF with promising results. HCA consists of a subxiphoid, surgical ablation followed by completion endocardial ablation. This meta-analysis of randomized control trials (RCT’s) and propensity score-matched studies aims to examine the efficacy and safety of HCA compared to endocardial catheter ablation (ECA) alone on patients with AF.

**Methods:**

This review was written in accordance with preferred reporting items for systematic reviews and meta-analyses recommendations and guidance. The primary outcome for the analysis was freedom from AF (FFAF) at final follow up. Secondary outcomes were mortality and significant complications such as tamponade, sternotomy, esophageal injury, atrio-esophageal fistulae post procedurally.

**Results:**

Four studies where included, with a total of 233 patients undergoing HCA and 189 patients undergoing ECA only. Pooled analysis demonstrated that HCA cohorts had significantly higher rates of FFAF than ECA cohorts, with an OR of 2.78 (95% CI 1.82–4.24, P < 0.01, I^2^ = 0). Major post-operative complications were observed in significantly more patients in the HCA group, with an OR of 5.14 (95% CI 1.70–15.54, P < 0.01). There was only one death reported in the HCA cohorts, with no deaths in the ECA cohort.

**Conclusion:**

HCA is associated with a significantly higher FFAF than ECA, however, it is associated with increased post-procedural complications. There was only one death in the HCA cohort. Large RCT’s comparing the HCA and ECA techniques may further validate these results.

**Supplementary Information:**

The online version contains supplementary material available at 10.1186/s13019-022-01930-7.

## Introduction

Atrial fibrillation (AF) is the most common cardiac arrythmia, affecting approximately 33 million people worldwide [1]. Only 3% of all AF is associated with concomitant cardiac disease [2]. Endocardial catheter ablation (ECA) remains the mainstay of intervention in AF, however, its success may be limited by its inability to create transmural endocardial ablation lines [3–5]. Surgical ablation was initially developed with cut and sew lesions, first described by Cox et al. in 1991 [6]. With the advent of thoracoscopic radiofrequency devices, surgical treatment of AF has shifted from open heart to minimally invasive procedures to isolate the pulmonary veins, occlude the left atrial appendage and create epicardial ablation lines [5].

The success rate of a surgical approach is superior to that of ECA in persistent AF [7]. The advantage of the surgical approach is the ability to visualize and target the left atrial structures, especially the posterior wall [8]. There are limitations of endocardial approaches. Anatomically, the posterior wall is difficult to isolate from the endocardial approach [8]. Furthermore, extensive endocardial ablation risks thermogenic damage to surrounding structures such as the phrenic nerve or esophagus [5]. Strengths of endocardial ablation is its ability to map AF substrates and develop electrophysiological (EP) endpoints. Hybrid convergent ablation (HCA) garners the strengths of both approaches by combining a subxiphoid (surgical) approach to target the posterior left atrial wall, followed by completion endocardial ablation [8]. The ability to ablate the posterior wall, validate these lesions, identify further lesions and ablate further arrhythmogenic substrates makes this approach effective for rhythm maintanence [5]. Unfortunately, much of the convergent experience comes from single-centre retrospective studies which are prone to bias [5, 9].

The primary aim of this study is to assess the freedom from AF in a HCA cohort compared to an ECA cohort alone. The secondary aim of this study is to assess the incidence of significant complications such as tamponade, sternotomy, esophageal injury, atrio-esophageal fistulae post procedurally and post procedural mortality. In order to account for bias associated with retrospective studies, we included randomized control trials (RCT) and propensity score-matched (PSM) analyses only.

## Methods

### Literature search strategy

This review was undertaken in accordance with Preferred Reporting Items for Systematic Reviews and Meta-Analyses (PRIMSA) recommendations and guidance [10]. Four electronic databases were used to perform the literature searches, including EMBASE, MEDLINE, PubMed and SCOPUS. These databases were searched from the date of database inception through to February 2022. A search strategy using a combination of keywords and Medical Subject Headings (MeSH) including “Hybrid Ablation” OR “Convergent procedure” AND “Atrial Fibrillation” AND “Outcomes” was carried out.

### Selection criteria

Studies were eligible for inclusion for this systematic review if (1) they compared HCA procedures to ECA only, (2) they reported freedom from AF, (3) were a RCT or PSM analysis (4), and were published in English. HCA was defined as a subxiphoid ablation followed by a catheter-based ablation either in the same sitting or staged sitting. Studies were excluded if patients underwent concurrent cardiac surgery, as well as conference abstracts, case reports, editorials, reviews and expert opinions. Article identification and inclusion were performed independently by two authors (AE and ARWS) and discussed until consensus was reached. A third author (CDF) resolved any conflicts. Study quality was subsequently assessed utilising the Delphi Criteria [11].

### Outcomes of interest and data extraction

The primary outcome for the analysis was freedom from AF (FFAF) at final follow-up. This was defined as per the study methodology (see above). Secondary outcomes were significant complications post-procedurally. These include, but were not limited to, death, tamponade, emergency sternotomy, pericardial hernia, phrenic nerve palsy, stroke and esophageal injury. Baseline variables including age, gender, pre-procedural duration of AF, left ventricular ejection fraction (LVEF), left atrial (LA) size were aggregated. An ad-hoc analysis was conducted and extraction was performed with analysis of all texts, figures, tables, and supplementary data. This was performed independently by two authors (AE and ARWS) until consensus was reached for final data.

### Statistical analysis

For baseline variables, nominal data was recorded as the number of events (n) and expressed as a percentage. Continuous variables were expressed as mean and standard deviation, or median and interquartile ranges. Baseline patient data were aggregated. Medians and interquartile ranges were first converted to mean and standard deviation utilizing the method outlined by Hozo et al. [12]. Significant differences in means and proportions were calculated using STATA (Version 17.0, StataCorp, Texas, USA). Meta analysis was carried out on Review Manager 5 (RevMan5®, Version 5.3, Copenhagen: The Nordic Cochrane Centre, The Cochrane Collaboration 2014). Due to the varied patient population, a random effects model was chosen for all analyses. Heterogeneity was assessed using the I^2^ test statistic. Low heterogeneity was denoted by I^2^ < 50%, moderate heterogeneity by I^2^ 50–74%, and high heterogeneity by I^2^ ≥ 75%. Publication bias was further assessed with an influential study analysis using the leave one out method. P vales less than 0.05 were deemed significant. Kaplan–Meier survival curves were digitized where presented and an algorithmic computational tool was utilized to derive individual patient data as outlined by Guyot et al. [13]. Event and censoring data were compiled for 5 years, and overall survival curves were produced utilizing Stata®.

## Results

### Search results and operative characteristics

Our search produced a total of 812 articles, of which 349 duplicates were removed. 463 abstracts were screened and 18 studies were selected for full text review. Four studies were selected for inclusion [14–17]. The search strategy is summarized in Additional file 1: Figure 1. Two studies were RCT’s [15, 17] and two studies were PSM analyses [14, 16]. The number of patients varied from 50 to 153. All studies provided inclusion criteria for patients, three of which included patients with persistent or longstanding AF and one study paroxysmal AF.

All four studies utilized a subxiphoid transdiaphragmatic approach in the HCA arm. In terms of lesion sets, all four studies conducted parallel/linear lesions on the posterior left atrial wall, followed by pulmonary vein isolation in the catheter arm. All four studies also utilised additional endocardial ablation lines, and utilised electroanatomic mapping to guide lesion sets. Three studies [14–16] defined recurrence as “an episode of AF lasting greater than 30 s” and one study defined recurrence an AF burden greater than 1% of the time [17]. All four studies utilised continuous monitoring during follow up for AF recurrence. These results are summarized in Additional file 1: Table 1.

### Baseline patient data

A total of 233 patients underwent HCA and 189 patients underwent ECA for AF. The mean age for the HCA and ECA cohorts were comparable, at 63.7 years and 63.4 years respectively (P = 0.77). There were more males in the HCA cohort (185 vs. 127 P = 0.045). There were no differences in BMI between the cohorts, with a mean BMI of 33.3 for the HCA group and 33.7 for the ECA group (P = 1.00). The mean preoperative EF was 54.9% for the HCA group and 54.6% for the ECA group (P = 0.97). Left atrial size were also comparable, with a mean diameter of 45.9 mm in the HCA group and 46.1 mm in the ECA group (P = 0.70) (Table 1).

**Table 1:** Baseline patient variables

Authors	DCCV hybrid	DCCV catheter	Mean age hybrid	Mean age catheter	Male hybrid	Male catheter	Mean AF hybrid	Mean AF catheter	BMI hybrid	BMI catheter	LVEF hybrid	LVEF catheter	LA diameter hybrid	LA diameter catheter
Kress et al	51 (80%)	53 (77%)	60.7	62.3 (8)	59 (80%)	51 (74%)	12	7	35	34.6	53.6	53.4	48	47
Maclean et al	28 (65%)	22 (51%)	68.6	65.5	30/43	28 (65%)	36	28	NR	NR	50%	50%	47.4	47.5
DeLurgio et al	2.0 ± 1.1*	3 ± 2.3*	63.7	65.1	80/102	27 (53%)	NR	NR	32.9	35.1	55.3	55.7	44	43
Jan et al	1.1 ± 1.8*	1.4 ± 1.8*	58.8	59.5	16/24	21 (81%)	49.2	62.4	30.6	29	65.55	63.33	32.4**	34.2**

Direct Current Cardioversion (DCCV)/Body Mass Index (BMI)/Left Ventricular Ejection Fraction (LVEF)/Left Atrial (LA)

*Mean ± SD within the last 12 months

**Volume (mls)

### Primary outcome

**Fig. 1:**
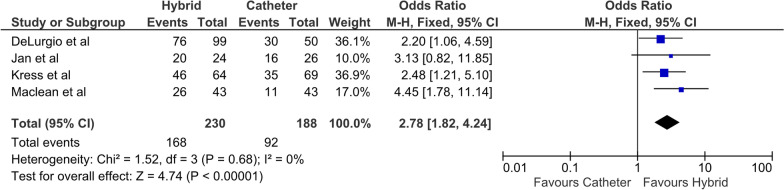
Primary outcome (freedom from AF)

**Fig. 2:**
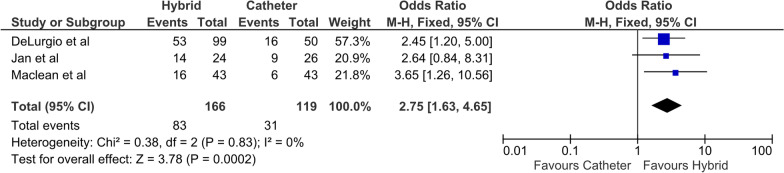
Freedom from AF off AAD

**Fig. 3:**
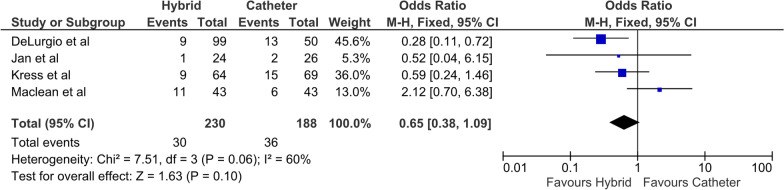
Subsequent DCCV during follow up

All four studies reported FFAF, with 73% and 49% in the HCA and ECA cohorts at last follow-up, respectively. The odds ratio (OR) was 2.78 (95% CI 1.82–4.24, P < 0.01) (Fig. 1) favoring HCA. There was low heterogeneity between the studies (I^2^ = 0). All four studies reported a significantly greater freedom from AF in the HCA cohort. Three studies reported freedom from AF off anti-arrhythmic drug therapy (AAD) with 50% and 26% in the HCA and ECA cohorts, respectively**.** The OR was 2.75 (95% CI 1.63–4.65, P < 0.001) (Fig. 2), favoring HCA [14, 15, 17]. All three studies demonstrated a greater degree of freedom from AF off AAD in the HCA group. All four studies reported additional direct current cardioversion (DCCV) procedures during follow up, with 13% and 19% of HCA and ECA cohorts, respectively. This did not reach significance and was associated with moderate heterogeneity (I^2^ = 60%) (Fig. 3).

### Secondary outcomes

**Fig. 4:**
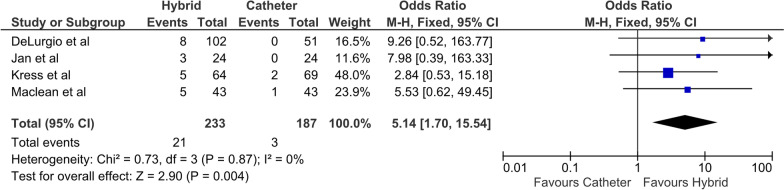
Secondary outcome (post-operative morbidity)

Secondary outcomes were reported in all four studies. Significantly more post-operative complications were observed in patients from the HCA group, with an OR of 5.14 (95% CI 1.70–15.54, P < 0.01) (Fig. 4). There were no deaths in the ECA cohort and one death in the HCA cohort. Two patients in the HCA cohort sustained cardiac injuries requiring urgent sternotomy. Of patients who underwent HCA, 7 patients had a postoperative pericardial effusion. Four patients had significant postoperative bleeding, of which three required a sternotomy. Phrenic nerve palsy was observed in three patients. There were no reports of atrioesphageal fistulas or esophageal injuries in either cohort. Five of the 21 complications observed in the HCA cohort occurred after the endocardial component. Two patients had groin related complications requiring intervention [16]. Two patients had pericardial effusions requiring drainage and one patent sustained a phrenic nerve palsy after the endocardial component of HCA [14]. Significant post-procedural complications were observed in three patients in the ECA cohort. The breakdown of complications are summarised in Additional file 1: Table 2a and 2b. Primary and secondary outcomes are summarised in Table 2.

**Table 2:** Primary and secondary outcomes

Study	Year	follow up	# hybrid	# Catheter	Freedom from AF hybrid	Freedom from AF catheter	Primary outcome off AAD hybrid	Primary outcome off AAD catheter	Repeat DCCV hybrid	Repeat DCCV catheter	Death hybrid	Death catheter	Secondary outcome hybrid	Secondary outcome catheter
Kress et al.	2017	Median 16 months	64	69	46/64	35/69	NR	NR	9/64	15/69	1	0	5/64	2/69
Maclean et al.	2020	12 months	43	43	26/43	11/43	16/43	6/43	11/43	6/43	0	0	5/43	1/43
DeLurgio et al.	2020	12 months	102	51	76/99	30/50	53/99	16/50	9/99	13/50	0	0	8/102	0/51
Jan et al.	2018	Mean 30.5 months	24	26	20/24	16/26	14/24	9/26	1/24	2/26	0	0	3/24	0/24

### Aggregate data

**Fig. 5:**
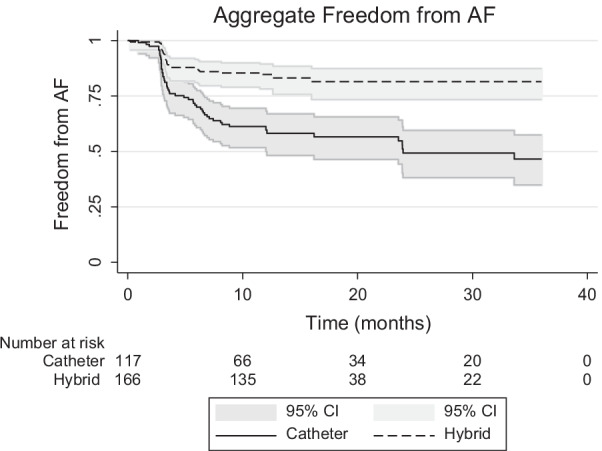
Aggregate freedom from AF

All four studies provided Kaplan–Meier (KM) curves, however, only three studies provided numbers at risk and therefore only these were included in the aggregate survival analysis. Actuarial survival (FFAF) at intervals of 6, 12, 18, 24 and 30 months were 87%, 85%, 83%, 83% and 83% for the HCA cohort, compared to 70%, 61%, 57%, 50%, 50% for the ECA cohort (Fig. 5).

### Study quality and Bias

Overall study quality ranged from average to good, with three studies deemed good and one study deemed average when assessed using the Delphi criteria (Table 3). As patients were either matched or randomized, systematic bias and variability in preoperative variables were minimal. A leave one out analysis was conducted to assess the impact of influential studies. As such, there was no significant effect on either the effect size or heterogeneity.

**Table 3:** Study quality utilising the 8-point Delphi criteria

Study	D1	D2	D3	D4	D5	D6	D7	D8	D9	Overall
Maclean et al.	Low	Low	High	High	Low	Low	Low	High	Average	High
Kress et al.	Low	Low	Average	High	Low	low	low	High	Average	Average
DeLurgio et al.	High	Low	High	High	Low	Low	Low	High	High	High
Jan et al.	High	Low	High	High	Low	Low	Low	High	High	High

## Discussion

This systematic review and meta-analysis of RCT’s and PSM studies aims to compare the efficacy of HCA to ECA. The inclusion of unmatched retrospective analyses into meta-analysis adds a significant degree of heterogeneity. Firstly, these studies have inherent patient related bias. Their retrospective design means inability to control for baseline variables that impact procedural efficacy, principally, number of previous ablations, LA dimensions, BMI, duration of AF and use of AAD [19–22]. Secondly, patients who undergo hybrid procedures are more likely to have complex arrhythmogenic substrates, have failed previous ablations, and are therefore more resistant to further ablations [19, 21, 22]. As a result, the effectiveness of HCA may be underestimated in these studies. Lastly, previous meta-analyses included studies with a variety of surgical techniques (subxiphoid/ unilateral or bilateral thoracoscopic/mini-thoracotomy/sternotomy), which vary in terms of their efficacy and risk profile [9, 18, 20, 22]. The resultant heterogeneity in these meta-analyses was significant [8, 22]. Mhanna et al. reported a heterogeneity of 77% in their primary outcome (freedom from AF at last follow-up), and Zhang et al. reported a heterogeneity of 86% in the same outcome [9, 18]. A strength of the present study was the inclusion of RCT’s and PSM studies only. All patients underwent the same procedure and had similar pre-operative characteristics. The resultant heterogeneity was significantly smaller than previously published meta-analyses (I^2^ = 0) for the primary outcome of interest.

The FFAF in the HCA cohort was significantly higher than the ECA cohort and is higher than the FFAF in previously published meta-analyses. Mhanna et al. reported a FFAF of 70% with an OR of 1.48 (95%CI 1.13–1.94, P < 0.01) favoring HCA [8]. Zhang et al. reported a FFAF in their HCA cohort of 57%, with an OR of 2.10 (95% CI 0.45–9.88), however, this failed to reach significance [18]. The main cohort of patients undergoing HCA in this study as well as previous meta-analysis was persistent AF, and the higher freedom from AF highlights the efficacy of HCA in this cohort.

Three studies reported FFAF off AAD. Subgroup analysis suggests that FFAF was 50% in the HCA cohort compared to 26% in the ECA cohort. Accordingly, reintervention rates were also low with HCA cohorts less likely to require subsequent DCCV. Some studies also suggest that HCA cohorts are less likely to require further ECA in the future, however this data was not reported by all included studies [17]. Our study also demonstrated a significantly lower attrition in FFAF in the HCA cohort versus ECA cohort, with an aggregate FFAF of 85% versus 61% at 12 months. The use of AAD can be associated with long-term side effects and cessation of use is beneficial. The continued decline of FFAF in ECA cohorts off AAD can be attributed to incomplete pulmonary vein isolation (PVI) or failure to address lesions that lie outside the PVI, thereby relying on AAD to maintain sinus rhythm [17]. The transmural lesions created by a hybrid convergent approach account for the higher FFAF off antiarrhythmic therapy, reduced need for further cardioversions and lower attrition rate over time observed in this study.

Three studies performed the HCA in the same sitting, and one study performed a staged HCA. Varzaly et al. performed a meta-analysis of rhythm maintenance following hybrid ablation, and found no significant differences between a staged or sequential approach [23]. Advantages of a sequential approach are immediate identification of lesion gaps that can be corrected by catheter ablation and shorter procedural times [23]. Advantages of a staged approach is that it allows time for lesions to mature and edema to regress, identifying definite lesions of further endocardial ablation 23. There is a paucity of literature exploring the utility of a staged procedure compared to a concomitant procedure, and future RCT’s are being conducted in the area to address this question.

Indication for AF in three studies was persistent AF, and one study performed convergent procedures on patients with paroxysmal AF. The recurrence rate for AF is usually dependent on the duration of AF, as the pathological mechanisms for persistent AF and long-standing persistent AF are more complex than paroxysmal AF [24]. The approach of PVI may not be effective enough for longstanding AF and further ablations are necessary. Patients may also need to undergo repeated procedures exposing them to the deleterious effects of radiation exposure, complications and cost [24]. The major strategic advantage of HCA over catheter ablation alone is the ability to attain a broad area of ablation across the entire posterior left atrial wall [25, 26]. PVI alone may not be sufficient in persistent AF as substrates are more likely to be located in non-pulmonary vein regions [25, 26]. Furthermore, the left atrial appendage, a further substrate for AF, can be closed with an additional thorascopic port [5].

Complications were more common in HCA cohort, with 9.4% of patients the HCA cohort and 1.6% in the ECA cohort reporting a post-procedural adverse event. Overall, mortality was rare, reported in only one patient (0.005%) in the HCA cohort secondary to a gastrointestinal bleed. Varzaly et al. reported a similar overall complication rate across 22 studies of 6.5%, with a low mortality rate of 0.2% reflecting the overall safety of the procedure [23]. Overall mortality is low in literature, with only one study documenting a high mortality rate [19]. The HCA group in that study experienced three deaths (in 24 patients) and these sudden deaths were attributed to the type of technology used (unipolar ablation) and the approach (pericardioscopic) [19]. The unipolar device has since been redesigned with an electrocardiogram sensing tip and an irrigation tip to reduce the rate of complications [4]. As HCA is an evolving field, future peri- and post-operative protocols may result in a lower complication rate. Delurgio et al. commented that all four postoperative effusions were potentially avoidable with postoperative non-steroidal therapy [15].

Cessation of oral anticoagulation therapy (OAC) post procedurally is an important outcome as the bleeding risk on OAC is not negligible. Themisocclatis et al. observed a 2% risk of major haemorrhage in patients on warfarin following catheter ablation [27]. The cessation of OAC depends on the maintenance of SR post procedurally and the CHADS-VASC2 score. The cessation of OAC post catheter ablation has been well studied. A meta-analysis by Liu et al. demonstrated a similar cumulative thromboembolic rate on and off OAC post ablation of 1.1% and 1.4% respectively [28]. Additionally, the rate of haemorrhagic complications in the group off OAC was significantly lower [28]. Convergent ablation hafs a higher FFAF than catheter ablation alone, expectedly the cessation of OAC would be higher in these cohorts. There is a paucity of evidence exploring this. Studies assessing post procedural success of minimally invasive surgical ablation demonstrate that the prevalence of OAC use was higher in the surgical cohorts, however this was confounded by patient bias whereby surgical cohorts have larger LAA dimensions and CHADS-VASC2 scores [29]. Lauritzen et al. demonstrated that OAC’s can be safety ceased 12 months post-surgical ablation, when patients have SR maintenance and a CHADS-VASC2 score less than 2 p^30^]. Future RCT’s exploring OAC cessation after convergent ablation would be beneficial.

There is also a paucity of evidence exploring the efficacy of convergent ablation in high-risk patients such as those with a high BMI and a previous history of cardiac surgery. Obese patients face greater risks of complications from ablation procedures due to their comorbidities p^31^]. During hybrid ablation, haemodynamic intolerance, stroke risk and ventilation may pose an issue [31]. Additionally, procedure times are usually longer and radiation exposure greater in obese patients [31]. Furthermore, the presence of epicardial fat may attenuate the energy delivered to the left atrial wall, diminishing the effectiveness of the procedure. These difficulties have not translated to a lower FFAF following thorascopic ablation [31, 32]. Patients with a previous history of cardiac surgery may have adhesions limiting exposure of the left atrium, and in which case the procedure may not be feasible. As the convergent procedure becomes more widespread and these cases are encountered, future cohort studies and RCT’s will investigate the issue further.

## Limitations

There were limitations to the present meta-analysis. A small number of studies, with small patient numbers, were included in the meta-analysis as a result of the inclusion of RCT’s and PSM studies only. Secondly, rhythm monitoring post-procedurally varied, with one study utilising an internal loop recorder and three utilising Holter monitoring and ECG’s. The definition of FFAF also varied between the studies, with three studies defining recurrence as 30 s of AF outside the blanking period and one study defining recurrence as an AF burden > 1% of the time. Again, this impacts the FFAF and adds systematic bias to the review. Finally, there was a paucity if individual patient data (IPD) to aggregate the KM curves past the one-year mark. Both Jan et al. and Kress et al. reported small patient numbers past the one year so deriving conclusions on AF free survival past the one-year mark is tenuous.

Future RCT’s, with larger patient cohorts and including LAA exclusion will further consolidate this data. As HCA is an emerging technology, trials with subgroup analysis assessing the advantage LAA exclusion or the vein of Marshall ablation are warranted. Also, pulsed field ablation as a part of convergent ablation is a novel technology with high efficacy, and future trials assessing this in larger patient populations will be beneficial [33].

## Conclusion

HCA is associated with a significantly higher FFAF than ECA alone and a higher likelihood of rhythm maintenance off AAD. HCA, however, is also associated with more post-procedural complications than ECA alone. The overall risk of mortality remains low. Future RCT’s with larger patient cohorts comparing the HCA and ECA may further validate these results.

## Supplementary Information

**Additional file 1: Supplementary Figure 1.** PRISMA flow-chart summarizing the search strategy for relevant publications. **Supplemental Table 1.** Operative Characteristics. **Supplemental Table 2a.** Postoperative complications (HCA). **Supplemental Table 2b.** Postoperative complications (ECA).

## Data Availability

The datasets used and/or analysed during the current study are available from the corresponding author on reasonable request.

## Abbreviations

AF: Atrial fibrillation
HCA: Hybrid convergent ablation
ECA: Endocardial catheter ablation
RCT: Randomized control trials
PSM: Propensity score-matched
PRIMSA: Preferred reporting items for systematic reviews and meta-analyses
FFAF: Freedom from AF
AAD: Anti-arrhythmic drug therapy
LVEF: Left ventricular ejection fraction
LA: Left atrial
DCCV: Direct current cardioversion
KM: Kaplan–Meier
PVI: Pulmonary vein isolation
OAC: Oral anticoagulation therapy
