# Supplementary material for: Hybrid convergent ablation versus endocardial catheter ablation for atrial fibrillation: a systematic review and meta-analysis of randomised control trials and propensity matched studies
Source: J Cardiothorac Surg. 2022 Aug 13;17:181. doi: 10.1186/s13019-022-01930-7 (PMC9375401; doi:10.1186/s13019-022-01930-7)
Supplement: Supplementary file 1 — Additional file 1: Supplementary Figure 1. PRISMA flow-chart summarizing the search strategy for relevant publications. Supplemental Table 1. Operative Characteristics. Supplemental Table 2a. Postoperative complications (HCA). Supplemental Table 2b. Postoperative complications (ECA). [file 13019_2022_1930_MOESM1_ESM.docx]

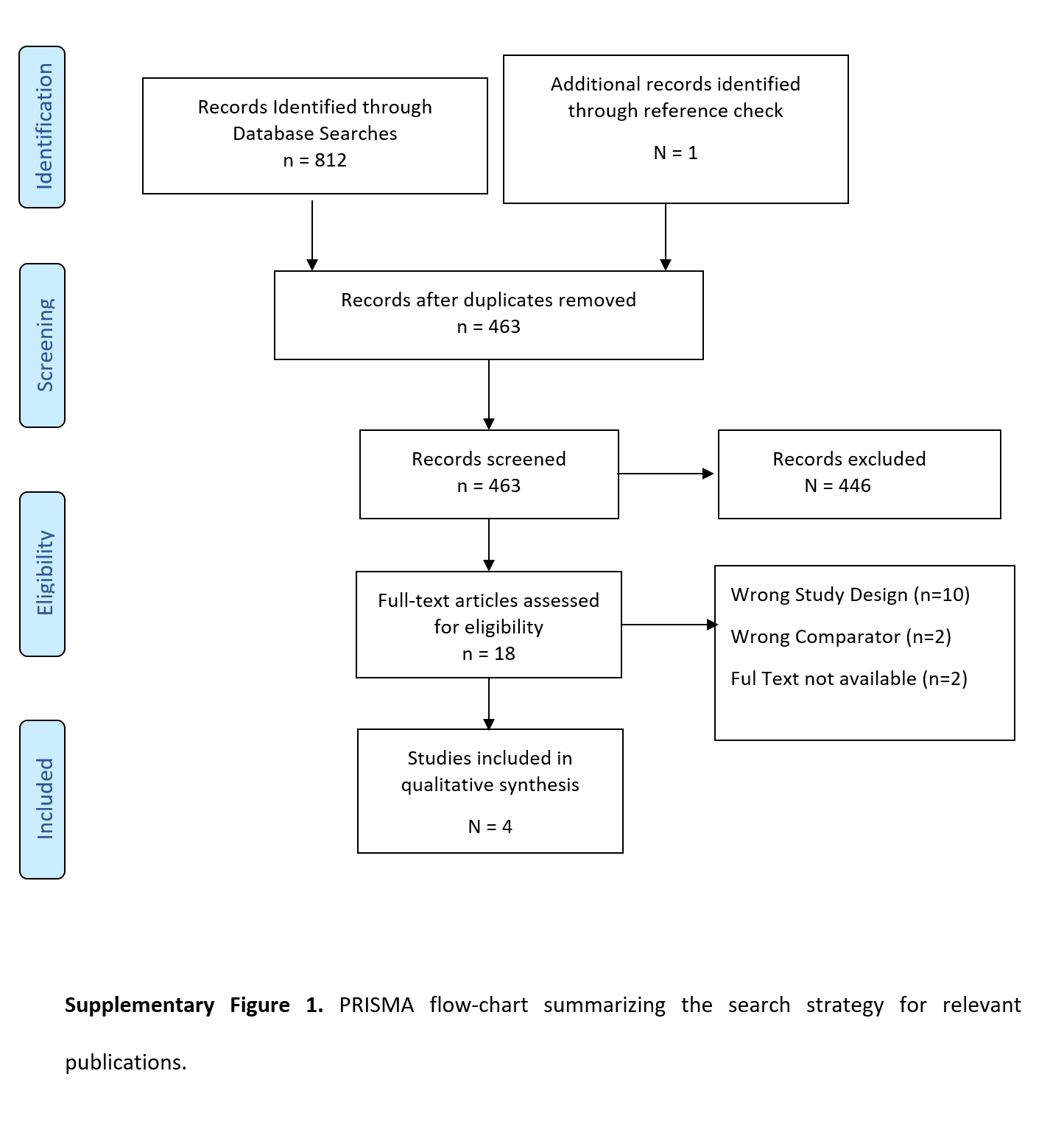


**Supplemental Table 1: Operative Characteristics**

| Study | Inclusion Criteria | Access | Device | Basic Lesion set surgical | Lesion set Catheter | Electro anatomical mapping? | Timing | Recurrent catheter ablations | Mean/ subsequent ablations | Procedure duration hybrid | Procedure duration catheter | Definition of recurrence | Postoperative follow up | Postoperative arrythmia measurement |
| --- | --- | --- | --- | --- | --- | --- | --- | --- | --- | --- | --- | --- | --- | --- |
| Maclean et al | Persistent AF (> 12 months) | Subxiphoid transdiaphragmatic | Atricure EPISense | Linear lesions posterior LA wall | PVI and additional posterior wall isolation  CFAE ablation (operator dependant), additional lines operator dependant | CARTO | Staged (6 weeks later) | Yes | NR | NR | NR | 30 seconds of documented AF outside the blanking period | 3,6,12 months | 72H Holter, ECG, Echocardiogram |
| DeLurgio et al | Persistent AF refractory or intolerant to AAD  LA size < 6 cm | Subxiphoid transdiaphragmatic | Atricure EPISense | Linear lesions Posterior LA wall  R and L PV antrum | PVI,  96% of patients cavo-tricuspid isthmus ablation,  All patients’ linear lesions connecting superior PV's, 26% and 4% CFAE in convergent/catheter arm respectively, 2% mitral isthmus line | Not specified | Same | Yes | NR | 178.7min | 171.4 min | Any episode of AF/AT greater than 30 seconds by Holter monitoring, use of new AAD and dosage increase of AAD, subsequent catheter ablation or cardioversion at any point in time | 6, 12, 18 months | 24H Holter, 7-day Holter |
| Kress et al | Persistent or Longstanding AF | Subxiphoid transdiaphragmatic | Atricure Numeris probe | Linear lesions posterior LA wall PV trunk | PVI. First 24 patients, RFA used to isolate pulmonary veins. RFA for CFAE's and/or linear lesions following PVI in all but 3 patients. | CARTO | Same | Yes | 6/18 | 313.7 min | 233.1 min | 30 seconds of AF on ECG, monitoring or telemetry, DCCV, Ablation and PPM insertion outside blanking period | 3,6,9,12,18 months | Multiple: 30-s electrocardiographic recording or rhythm strip, ambulatory monitoring, including pacemaker interrogation, loop recorder, ambulatory cardiac telemetry monitor, or Holter monitor |
| Jan et al | Paroxysmal AF | Subxiphoid transdiaphragmatic | Atricure EPISense | Linear lesions Posterior LA wall | PVI | Ensite NavX | Same | Yes | 4/9 | 266 min | 242 min | AF burden > 1% of time | 3, 6, 12 months | Internal Loop Recorder |

**Supplemental table 2a: Postoperative complications (HCA)**

| Study | Postoperative complications | TIA/Stroke | Excessive bleeding and/or sternotomy | Pericardial effusion | Phrenic nerve palsy | Pericardial Hernia | Complications due to endocardial component | Other |
| --- | --- | --- | --- | --- | --- | --- | --- | --- |
| Kress et al | 5 | 1 | 1^a^ | 1 | 0 | 0 | 2^b^ | - |
| Maclean et al | 5 | 0 | 1 | 2 | 1 | 1 | 3 | - |
| Delurgio et al | 8 | 2 | 1 | 4 | 1 | 0 | NR | - |
| Jan et al | 3 | 0 | 1 | 0 | 0 | 0 | 0 | 2c |
| 1. Gastrointestinal bleeding resulting in death 2. 2 groin complications form puncture site requiring intervention 3. One acute lung injury, one infection | | | | | | | | |

**Supplemental table 2b: Postoperative complications (ECA)**

| Study | Postoperative complications | TIA/Stroke | Excessive bleeding and/or sternotomy | Pericardial effusion | Phrenic nerve palsy | Atrio-oesophageal fistulae | Other |
| --- | --- | --- | --- | --- | --- | --- | --- |
| Kress et al^a^ | 2 | 0 | 0 | 2 | 0 | 0 | - |
| Maclean et al | 1 | 0 | 0 | 1 | 0 | 0 | - |
| Delurgio et al | 0 | 0 | 0 | 0 | 0 | 0 | - |
| Jan et al^b^ | 0 | 0 | 0 | 0 | 0 | 0 | - |
| 1. One patient sustained a TIA with rapid and full reversal of symptoms – termed a minor complication 2. One patient had a readmission with ventricular tachycardia, requiring resuscitation and DCCV and was not strictly procedurally related. | | | | | | | |
